# Epigenetic Alterations of Chromosome 3 Revealed by NotI-Microarrays in Clear Cell Renal Cell Carcinoma

**DOI:** 10.1155/2014/735292

**Published:** 2014-05-22

**Authors:** Alexey A. Dmitriev, Evgeniya E. Rudenko, Anna V. Kudryavtseva, George S. Krasnov, Vasily V. Gordiyuk, Nataliya V. Melnikova, Eduard O. Stakhovsky, Oleksii A. Kononenko, Larissa S. Pavlova, Tatiana T. Kondratieva, Boris Y. Alekseev, Eleonora A. Braga, Vera N. Senchenko, Vladimir I. Kashuba

**Affiliations:** ^1^Engelhardt Institute of Molecular Biology, Russian Academy of Sciences, Moscow 119991, Russia; ^2^P.A. Herzen Moscow Oncology Research Institute, Ministry of Healthcare of the Russian Federation, Moscow 125284, Russia; ^3^Institute of Molecular Biology and Genetics, Ukrainian Academy of Sciences, Kiev 03680, Ukraine; ^4^Mechnikov Research Institute for Vaccines and Sera, Russian Academy of Medical Sciences, Moscow 105064, Russia; ^5^National Cancer Institute, Kiev 03022, Ukraine; ^6^N.N. Blokhin Russian Cancer Research Center, Russian Academy of Medical Sciences, Moscow 115478, Russia; ^7^Institute of General Pathology and Pathophysiology, Russian Academy of Medical Sciences, Moscow 125315, Russia; ^8^Research Center of Medical Genetics, Russian Academy of Medical Sciences, Moscow 115478, Russia; ^9^Department of Microbiology, Tumor and Cell Biology, Karolinska Institute, 17177 Stockholm, Sweden

## Abstract

This study aimed to clarify epigenetic and genetic alterations that occur during renal carcinogenesis. The original method includes chromosome 3 specific NotI-microarrays containing 180 NotI-clones associated with 188 genes for hybridization with 23 paired normal/tumor DNA samples of primary clear cell renal cell carcinomas (ccRCC). Twenty-two genes showed methylation and/or deletion in 17–57% of tumors. These genes include tumor suppressors or candidates (*VHL, CTDSPL, LRRC3B, ALDH1L1*, and *EPHB1*) and genes that were not previously considered as cancer-associated (e.g., *LRRN1, GORASP1, FGD5*, and *PLCL2*). Bisulfite sequencing analysis confirmed methylation as a frequent event in ccRCC. A set of six markers (*NKIRAS1/RPL15, LRRN1, LRRC3B, CTDSPL, GORASP1/TTC21A*, and *VHL*) was suggested for ccRCC detection in renal biopsies. The mRNA level decrease was shown for 6 NotI-associated genes in ccRCC using quantitative PCR: *LRRN1, GORASP1, FOXP1, FGD5, PLCL2,* and *ALDH1L1*. The majority of examined genes showed distinct expression profiles in ccRCC and papillary RCC. The strongest extent and frequency of downregulation were shown for *ALDH1L1* gene both in ccRCC and papillary RCC. Moreover, the extent of *ALDH1L1* mRNA level decrease was more pronounced in both histological types of RCC stage III compared with stages I and II (*P* = 0.03). The same was observed for *FGD5* gene in ccRCC (*P* < 0.06).

*Dedicated to thememory of Eugene R. Zabarovsky*

## 1. Introduction

Renal cell carcinoma (RCC) has the highest mortality rate of the genitourinary cancers [1]. Each year about 190 000 new renal cancer cases are diagnosed and about 90 000 men die worldwide [2]. Generally RCC tend not to cause symptoms in early stages, whereas in patients with more advanced disease symptoms are nonspecific. More than 60% of RCC are detected incidentally during diagnostic tests (ultrasound, computed tomography, etc.). About a third of patients with RCC already have locally advanced or metastatic disease. Patients with metastatic RCC have a median survival of around 13 months and the 5-year survival rate is under 10% [1]. RCC is represented by several histological types. The three most common of them are clear cell RCC (ccRCC, 75–80% of cases), papillary RCC (pRCC, 10–15% of cases), and chromophobe RCC (5% of cases). Both ccRCC and pRCC are derived from the common origin—proximal convoluted tubule [2].

Renal cancer is characterized by numerous genetic and epigenetic alterations [1]. DNA methylation is a key epigenetic mechanism that is known to be precisely regulated during cell differentiation and plays a crucial role in the control of gene expression and in cancer [3]. Hypermethylation of promoter region of genes, primarily tumor suppressor genes (TSGs), and hypomethylation of other genome elements are common events in cancer [4].

Hemizygous deletion of several regions of chromosome 3p (short arm of chromosome 3) and inactivation of TSG* VHL* are the most common genetic alteration in ccRCC [5]. The important role of chromosome 3 in cancer is well known; its short arm harbors several regions that include many known tumor suppressor genes and TSG-candidates [6, 7]. But a comprehensive analysis of methylation status of chromosome 3 in ccRCC was still not performed.

Recently, by Eugene R. Zabarovsky, a sensitive technology based on NotI-microarrays (NMA) was developed for identification of both genetic (deletions/amplification) and epigenetic (methylation/demethylation) changes simultaneously. This technology was successfully used for analysis of methylations/deletions in lung [8], ovary [9], and cervical cancer [10]. NMA's methodology has been described in detail earlier [9, 11]. The essence of this method consists of the ability of the NotI-restriction enzyme to recognize and digest only the unmethylated motif 5′-GCGGCCGC-3′ often found in CpG-islands. CpG-islands are located in promoter region of many genes associated with cancer and its hypermethylation has been observed as a frequent mechanism of TSGs inactivation, which contributes to malignant transformation [4]. High sensitivity and specificity of the hybridization method were achieved by a special procedure of isolation of genomic DNA fragments flanking NotI-digested sites from total tumor/normal DNA. Thus, only a small fraction (0.05–0.10%) of the human genome is labeled and is used as a CpG-enriched probe for comparative hybridization [12]. Decreased hybridization signal of tumor DNA compared with normal DNA indicates the methylation and/or deletion of NotI-associated DNA fragments, whereas increased signal suggests the amplification and/or demethylation.

The aim of our study was to identify epigenetic and genetic alterations of chromosome 3 genes in ccRCC. Obtained data will help to clarify molecular mechanisms of ccRCC and provide a wide area for further investigations. Identified genes could be TSG-candidates and potential markers.

## 2. Materials and Methods

### 2.1. Tissue Specimens

Paired specimens of renal cancer tissues including 23 clear cell renal carcinomas, 12 papillary renal cell carcinomas, and morphologically normal tissues (conventional “normal” tissues) were obtained after surgical resection prior to radiation or chemotherapy and stored in liquid nitrogen. Sample information is represented in Table 1. The diagnosis was verified by histopathology and only samples containing 70–80% or more tumor cells were used in the study. “Normal” controls were obtained minimally at 2 cm distance from the tumor and confirmed histologically as normal epithelial cells. Tumor specimens were characterized according to the International System of Classification of Tumors, based on the tumor-node-metastasis (TNM) and staging classification of the Union for International Cancer Control (UICC, version 2002) [13] and World Health Organization (WHO) criteria classification [2]. The study was done in accordance with the principles outlined in the Declaration of Helsinki (1964). Informed consent was obtained from all patients. All tissues were collected under the approval of The Ethics Committee of N.N. Blokhin Russian Cancer Research Center, Russian Academy of Medical Sciences.

### 2.2. DNA, RNA, and cDNA Preparation

Total RNA and DNA were isolated from tumor and conventional “normal” tissues using RNeasy Mini Kit (Qiagen, Germany) and DNA extraction kit (Invitrogen, USA) according to the instructions of manufacturers. Purified RNA was quantified using NanoDrop ND-1000 spectrophotometer (NanoDrop Technologies, USA), and the quality was determined by Bioanalyzer 2100 (Agilent Technologies, USA). All RNA samples were treated with DNase I (Invitrogene) and cDNA was synthesized using M-MuLV reverse transcriptase and random hexamers (Fermentas, Lithuania) according to the standard manufacturer's protocol.

### 2.3. NotI-Microarrays

NotI-microarrays contained 180 NotI-linking clones that were associated with 188 genes from human chromosome 3. NotI-linking clones with inserts up to 15 kb were immobilized on the glass slides in six replications each [11, 12]. Plasmid DNA for immobilization on the glasses was isolated with a HiPure Plasmid Midiprep kit (Invitrogen) and printed on the siliconized glasses at a concentration of 0.25 *μ*g/*μ*L with a QarrayMini microarrayer (Genetix, United Kingdom). DNA from* E. coli* was used as negative hybridization control. Preparation of NotI-probes was done essentially as described previously [9, 14] using modified oligonucleotides for NotI-linker: NotAntBio—5′-Biotin-CAGCACTGACCCTTTTGGGACCGC-3′ and NotAntComp—5′-GGCCGCGGTCCCAAAAGGGTCAGTGCTG-3′. Hybridization of NotI-probes was carried out at 42°C for 15 h in a Lucidea Base device (Amersham Pharmacia Biotech, United Kingdom) according to manufacturer's recommendations. Microarrays were scanned in a GenePix 4000 A (Amersham Pharmacia Biotech). The results were processed with GenePix Pro 6.0 software (Amersham Pharmacia Biotech). Then data were analyzed using our program NIMAN (NotI-Microarray Analysis) [8].

### 2.4. Bisulfite Genomic Sequencing

The bisulfite conversion of DNA was carried out using an EZ DNA Methylation Kit (Zymo Research, USA) according to the manufacturer's instructions. Primers for PCR are available upon request. After amplification of bisulfite-treated DNA, PCR products were cloned and used for automated sequencing on ABI Prism 3100-Avant Genetic Analyzer (Applied Biosystems, USA).

### 2.5. Quantitative PCR

QPCR was performed with Applied Biosystems commercial primer-probe sets (inventoried sets were used for all 8 target genes) using a 7500 Real-Time PCR System (Applied Biosystems). Each reaction was repeated three times. QPCR data were analyzed using two reference genes,* GUSB* and* RPN1* [15], and the relative quantification (ΔΔ*C*_*t*_) method. Relative mRNA level (*R*) was calculated by the following formula:

(1) $$\begin{array}{c} R=\frac{{\left(2^{\log_{2}(1+E^{\text{tar}})\times C_{t}^{\text{tar}}-\log_{2}(1+E^{\text{ref}})\times C_{t}^{\text{ref}}}\right)}_{\text{“normal”}\;\text{tissue}}}{{\left(2^{\log_{2}(1+E^{\text{tar}})\times C_{t}^{\text{tar}}-\log_{2}(1+E^{\text{ref}})\times C_{t}^{\text{ref}}}\right)}_{\text{cancer}\;\text{tissue}}}, \end{array}$$

where *E* is efficiency of reaction, *C*_*t*_ is replicate-averaged threshold cycle, ref is reference gene, and tar is target gene. All efficiencies were more than 90%. All calculations were performed using our program, ATG (analysis of transcription of genes) [16], compatible with Relative Quantification software (Applied Biosystems). At least 2-fold mRNA level changes were considered as significant.

### 2.6. Statistical Analysis

Nonparametric Wilcoxon test was used to compare mRNA expression differences of target and reference genes in renal cancer samples. Kruskal-Wallis and Mann-Whitney rank-sum tests, Fisher's exact test, and *χ* ^2^ criteria were used for analysis of methylation and mRNA level changes in renal cancer groups with different histological and pathological characteristics. *P* values < 0.05 were considered statistically significant (<0.06 for small samplings, which do not allow more precise evaluation). Set of markers for ccRCC identification was developed using support vector machine [17]. All statistical procedures were performed using our ATG [16] and NIMAN [8] software and BioStat software [18].

## 3. Results

### 3.1. Analysis of Methylation/Deletion Frequency of Chromosome 3 Genes in ccRCC Using NotI-Microarrays

**Figure 1:**
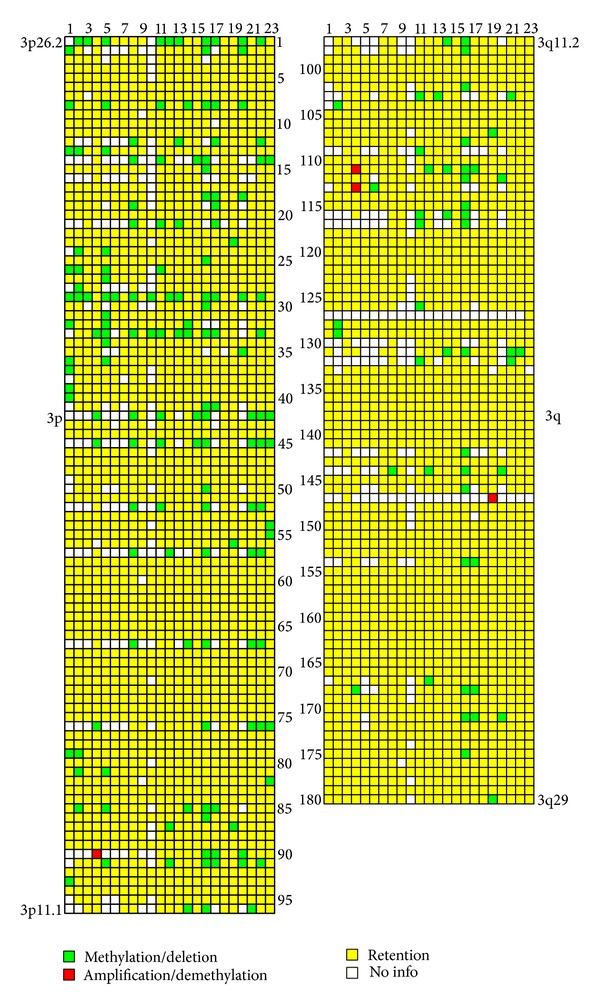
Pattern of DNA alterations in ccRCC. NotI-microarrays data. Vertically: 180 NotI-sites arranged according to their localization on chromosome 3 (from 3p26.2 to 3p11.1 and from 3q11.2 to 3q29). Horizontally: 23 ccRCC samples arranged by stage (from I to III). Green squares indicate methylation and/or deletion of DNA; red, amplification/demethylation; yellow, retention; and white, no information.

**Figure 2:**
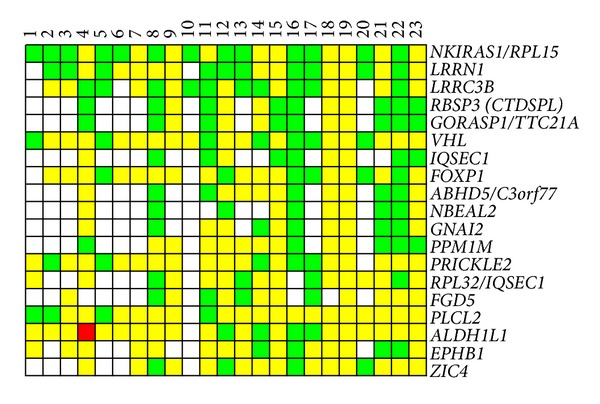
Pattern of DNA alterations in ccRCC for NotI-sites with high frequency of methylation and/or deletion. NotI-microarrays data. Vertically: 19 NotI-sites arranged by methylation/deletion frequency (from 57% to 17%). Horizontally: 23 ccRCC samples arranged by stage (from I to III), numbers correspond to numbers from Figure 1. Green squares indicate methylation and/or deletion of DNA; red, amplification/demethylation; yellow, retention; and white, no information.

The hybridization results of twenty-three NotI-enriched DNA probes from paired normal/tumor renal samples on chromosome 3 specific NMAs containing 180 NotI-linking clones that were associated with 188 genes are represented in Figure 1 and Supplementary Table 1 (see Supplementary Material available online at http://dx.doi.org/10.1155/2014/735292). As seen from Figure 1 methylation and/or deletions (M/D) were the main options of DNA alteration. Amplifications and/or demethylation represented single cases and were ignored in further analysis. The statistical analysis revealed 19 NotI-sites, associated with 22 genes, with methylation/deletion frequencies more than 17% (Figure 2, Table 2). Five out of 23 samples (22%) showed M/D simultaneously in more than 9 out of 19 NotI-sites with high M/D frequency and in at least 12 NotI-sites through whole NotI-microarray (Samples numbers 8, 11, 16, 17, and 22 from Figures 1 and 2). Among genes frequently methylated/deleted in ccRCC, only two were already known TSGs:* VHL* and* CTDSPL* (*RBSP3*). The majority of observed genes were previously not shown to be involved in renal carcinogenesis: among them were* LRRN1*,* GORASP1*,* FGD5*, and* PLCL2*.

### 3.2. Confirmation of NotI-Microarrays Results by Bisulfite Sequencing

**Figure 3:**
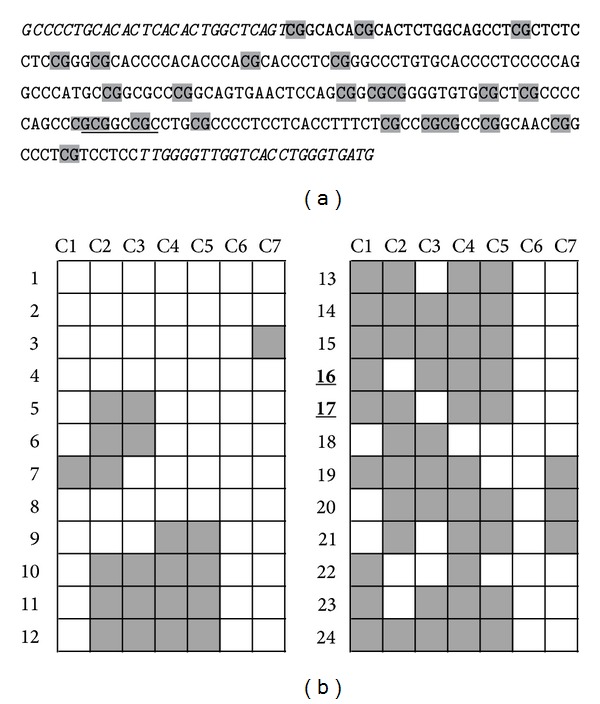
Methylation pattern of promoter CpG-island of* CTDSPL* gene in ccRCC. Bisulfite sequencing data for ccRCC sample number 8 from Figures 1 and 2 is represented. Twenty-four CpG-dinucleotides (a) are shown in grey. Primers for bisulfite sequencing (a) are marked in italic. NotI-site is underlined (a). In table (b) methylated (grey squares) and unmethylated (white squares) CpG-dinucleotides are shown for 7 sequenced clones. CpG-dinucleotides that correspond to NotI-site are marked in bold and underlined.

To confirm the results of NMA hybridizations, methylation status of promoter CpG-island of 5 genes with frequency of M/D 30–57% (according NMA) was analyzed:* NKIRAS1* (sample number 22 from Figures 1 and 2),* LRRN1* (numbers 5 and 17),* LRRC3B* (numbers 4 and 16),* CTDSPL* (numbers 8, 16, and 21), and* VHL* (numbers 1 and 5). Methylation was confirmed in all tested cases, except sample number 22 for* NKIRAS1* gene and sample number 1 for* VHL* gene. According to our previous qPCR data, deletions are the main mechanism of* NKIRAS1* gene inactivation in RCC [19], and deletion of* VHL* gene locus is a common genetic alteration in ccRCC [20]. Example of bisulfite sequencing data for* CTDSPL* gene is represented in Figure 3. As seen from Figure 3 dense methylation of promoter CpG-island was observed in the majority of sequenced clones. Thus, bisulfite sequencing data are in good concordance with NotI-microarrays results and suggest that methylation of 5′ regulator regions of genes is a frequent event in ccRCC.

### 3.3. Expression of* LRRN1, GORASP1, IQSEC1, FOXP1, GNAI2, FGD5, PLCL2*, and* ALDH1L1* Genes in ccRCC and pRCC

**Figure 4:**
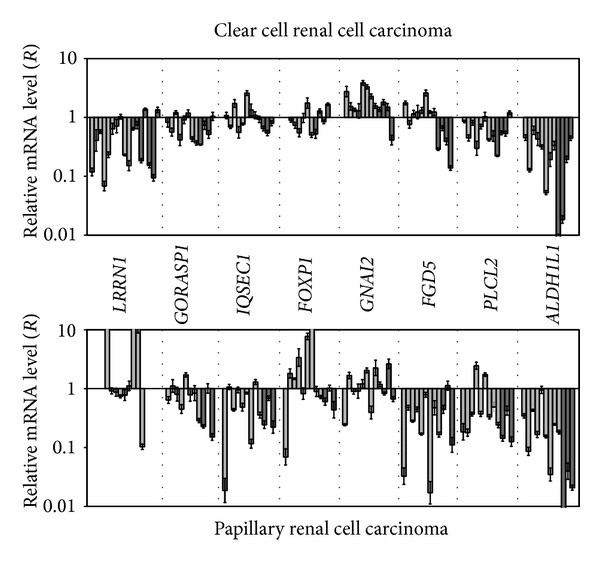
Relative mRNA level of 8 genes in ccRCC and pRCC. QPCR data. Light-grey bars correspond to tumors of stages I and II and dark-grey bars correspond to tumors of stage III.

Quantitative expression estimation was performed for eight genes showing high methylation/deletion frequency in ccRCC (*LRRN1, GORASP1, IQSEC1, FOXP1, GNAI2, FGD5, PLCL2*, and* ALDH1L1*; Figure 4). Six of them (except* IQSEC1 *and* GNAI2*) showed downregulation in 20–92% of ccRCC cases (Table 3). Expression level of* IQSEC1* gene was almost stable, and those of* GNAI2* even upregulated in 33% (4/12) of samples. The highest frequency and extent of the mRNA level decrease were observed for* LRRN1* and* ALDH1L1* genes (53% and 6-fold average decrease and 92% and 5-fold average decrease, resp.). In our previous study we showed downregulation of* NKIRAS1* gene in 75% (9/12) of RCC cases [19]. Two genes,* FGD5* and* ALDH1L1*, showed higher frequency and/or extent of downregulation in ccRCC of stage III than in ccRCC of stages I and II (*P* < 0.06 for* FGD5* gene).

The comparison of our NotI-microarray and qPCR data (Tables 2 and 3) showed that, for* LRRN1*,* GORASP1*,* FOXP1,* and* FGD5* genes M/D and downregulation, frequencies were close. In almost all cases (85%, 17/20) with detected M/D, the mRNA level was decreased. This allows us to assume that methylation and/or deletions were the main mechanisms of inactivation of these genes in ccRCC. For* PLCL2* and* ALDH1L1* genes, mRNA level decreased significantly more frequently than M/D were observed. This suggests the existence of other mechanisms of these genes' inactivation besides DNA methylation and/or deletions. On the contrary, for* IQSEC1* and* GNAI2* genes, no expression alteration or even upregulation was observed. This suggests additional mechanisms of these genes' activation, via miRNA, for example.

To compare expression alterations of the selected eight genes with high M/D frequency in two most common and at the same time morphologically distinct histological types of renal cancer the relative mRNA level in 12 pRCC samples was evaluated (Figure 4, Table 3). Expression profiles were different in pRCC and ccRCC for the majority of these genes. Only for* ALDH1L1* gene high frequency and extent of the mRNA level decrease were observed in both histological types of RCC. As well as in ccRCC, this gene showed stronger downregulation in pRCC of stage III than in pRCC of stages I and II (*P* = 0.03). On the whole, in pRCC five genes (*GORASP1*,* IQSEC1*,* FGD5*,* PLCL2,* and* ALDH1L1*) showed downregulation in 36–92% of cases. Thus, despite distinct expression profiles in pRCC and ccRCC, the decrease of mRNA level was the prevalent event in most of cases in both histological types of RCC.

## 4. Discussion

In this study we revealed 22 genes from chromosome 3 with high frequency (17–57%) of methylation and/or deletion in ccRCC using sensitive method of DNA hybridization on NotI-microarrays. Among them there are well-known TSGs and TSG-candidates* VHL*,* CTDSPL*,* LRRC3B*,* ALDH1L1,* and* EPHB1*. But there are also a number of genes which have not been previously reported as involved in cancer development, such as* LRRN1*,* GORASP1*,* FGD5*, and* PLCL2*. However, according to our recent data many of them are involved in the development of non-small cell lung cancer [8], cervical cancer [10], and high-grade serous ovarian cancer [9]. Sixteen out of 19 NotI-sites with high M/D frequency in ccRCC were located on the short arm of chromosome 3 (3p). These findings suggest that genetic and epigenetic destabilization of genes at chromosome 3 (especially 3p) are common mechanisms of epithelial tumors development.

Proteins encoded by the identified genes are involved in signaling pathways and biological processes frequently affected during development and progression of different cancer types, PRICKLE2 in WNT pathway; EPHB1 in Ephrin-EphR pathway; VHL and GORASP1 in apoptosis; CTDSPL (RBSP3) in cell cycle regulation; GNAI2 in transmembrane signaling systems; FGD5 in regulation of actin cytoskeleton; NKIRAS1 potent regulator of NFkappaB activity; and FOXP1 transcription factor, that are involved in tissue-specific expression [21, 22]. However, for a part of the genes, functions of their proteins are still unknown,* LRRN1*,* LRRC3B,* and* C3orf77,* for example.

We have validated our NotI-microarray data for* NKIRAS1*,* LRRN1*,* LRRC3B*,* CTDSPL,* and* VHL* genes by bisulfite genomic sequencing and have revealed that DNA methylation of the tested genes took place in ccRCC. However, hypermethylation of these genes does not exclude hemizygous deletions at the same time. Our data are in concordance with numerous studies describing hemizygous deletions of chromosome 3p regions [23, 24]. For example, frequent deletions of* ABHD5* gene in ccRCC were firstly discovered in the recent work [24]. We observed M/D of* ABHD5* gene region in 22% of samples, and our result supports this finding.

The identification of molecular markers for cancer diagnostics and prognostics is the crucial goal of modern molecular oncology [25, 26]. Potential clinical relevance of ccRCC biomarkers based on DNA methylation was shown in numerous publications [27–29], but the development of universal set of such markers with both high sensitivity and specificity is still an actual problem [30]. Using obtained NotI-microarray data, we constructed the prediction model for identification of ccRCC in renal biopsies. For detection of ccRCC at all stages, including stage I, the most perspective set is comprised of six markers:* NKIRAS1*/*RPL15*,* LRRN1*,* LRRC3B*,* RBSP3* (*CTDSPL*),* GORASP1*/*TTC21A,* and* VHL*. If methylation and/or deletion were found in two or more of these markers, then samples would be recognized as ccRCC. The sensitivity of this set was 78% and its specificity 96%. Gini coefficient was localized over the range 0.60–0.98. Prediction power of the developed set should be checked on samplings of renal biopsies in future. Moreover these markers should be tested for diagnostic use on biological fluids. Methylation alterations are one of the earliest events occurring during the tumor cell transformation process, and the gene methylation biomarkers are one of the most effective and advantageous for the early stage cancer screening [31].

QPCR revealed that methylation and/or deletions considerably contribute to inactivation of the majority of examined genes (*LRRN1*,* GORASP1*,* IQSEC1*,* FOXP1*,* GNAI2*,* FGD5*,* PLCL2,* and* ALDH1L1*) in ccRCC. Expression profiles obtained for these 8 genes in ccRCC and pRCC in this study and in lung SCC and ADC in our previous study [8] were significantly distinct. In lung SCC, high frequency of downregulation (>70%) was observed for 5 genes (*IQSEC1, FOXP1, LRRN1*,* GNAI2,* and* FGD5*); in lung ADC and pRCC, for 3 genes each (*IQSEC1*,* FOXP1*,* GNAI2,* and* FGD5*,* PLCL2*, and* ALDH1L1,* resp.); and in ccRCC, for only 1 gene (*ALDH1L1*).

The only gene showing strong mRNA level decrease both in ccRCC and in pRCC was* ALDH1L1*. ALDH1L1 (aldehyde dehydrogenase 1 family, member L1) catalyzes the conversion of 10-formyltetrahydrofolate, NADP, and water to tetrahydrofolate, NADPH, and carbon dioxide. It belongs to the aldehyde dehydrogenase family and is responsible for formate oxidation in vivo [22]. Downregulation of* ALDH1L1* gene at the mRNA and protein levels was observed in hepatocellular carcinoma, pilocytic astrocytoma, liver cancer, and cancer cell lines (A549, HepG2, 293A, Du-145, PC-3) [32–34]. Epigenetic silencing was shown in lung adenocarcinoma, spleen cancer, liver cancer, hemangioma, hepatocellular carcinoma, and cancer cell lines (A549, HepG2, HCT116) [35]. Moreover, ALDH1L1 suppressed cell motility in NSCLC cell line A549 [36]. Our data contribute to the tumor suppressor role of ALDH1L1 in carcinomas.

It is worth noting that* FGD5* gene revealed increased frequency and extent of mRNA level decrease in late stages compared to early in ccRCC as well as in lung SCC (*P* < 0.06 in both cases). Involvement of this gene in carcinogenesis was shown by us for the first time. Clarification of the role of* FGD5* gene in renal and lung cancer progression is a goal for the nearest future.

Comparison of NotI-microarrays and qPCR data showed that M/D is important, but not the sole mechanism of examined genes inactivation. Another significant mechanism of mRNA level regulation is microRNA [37]. To reveal possible coregulation of 22 genes with high M/D frequency by microRNA, we performed the analysis of TagetScan database [38] using in-house tool miRStat. It revealed several pairs of genes that could be coregulated by common microRNAs:* NKIRAS1* and* ABHD5* (miRs: 17, 106b, 20a, 93, 519c-3p),* NKIRAS1* and* FGD5* (miRs: 93, 519d, 20b),* NKIRAS1* and* IQSEC1* (miRs: 106b, 17, 519d),* FGD5* and* ABHD5* (miRs: 519d, 17, 106b, 20a, 93, 124, 506), and* FGD5* and* IQSEC1* (miRs: 106b, 93, 20b, 519d, 373, 520e, 520a-3p, 302c, 372, 520f, 4469, 212). As seen from the results of analysis 3 miRNAs (miRs: 93, 106b, 519d) could regulate expression of 4 genes (*NKIRAS1*,* FGD5*,* IQSEC1*, and* ABHD5*) simultaneously. MiR-93 is oncogenic [39, 40], miR-106b could play a dual role [41, 42], and the same is true for miR-519d [43, 44]. Thus, these miRNAs could contribute to the expression level alterations of the examined genes. Their impact on gene expression in ccRCC is one of our research interests in the nearest future.

This study reasserts that NotI-microarrays are powerful tools for disclosing TSG-candidates and potential biomarkers and provides a basis for better understanding of mechanisms involved in development of ccRCC.

## 5. Conclusions

NotI-microarray analysis revealed 22 genes with methylation and/or deletion frequency of more than 17% in ccRCC. For the majority of these genes, involvement in renal cancer was shown for the first time. Further analysis of signaling pathways alterations taking into account these genes is of research interest. Bisulfite genomic sequencing data confirmed NotI-microarrays results and showed that methylation of chromosome 3 genes (especially short arm—3p) is a common event in ccRCC. QPCR analysis revealed frequent (20–92%) downregulation of 6 genes with high methylation/deletion frequency in ccRCC. For one of these genes,* ALDH1L1*, the positive correlation of its mRNA level decrease with ccRCC and pRCC stage was shown (*P* = 0.03). Obtained data allowed us to suggest the set of 6 markers for ccRCC identification in renal biopsies with accuracy 87%.

## Supplementary Material

Supplementary Table 1. Summary of hybridization data of DNA from 23 clear cell renal cell carcinoma samples on chromosome 3 specific NotI-microarrays.

## Figures and Tables

**Table 1:** Pathological and histological characteristics of tumors.

Stage/TNM	Number of samples	
	ccRCC	pRCC
I/T_1_N_0_M_0_	7	5
II/T_2_N_0_M_0_	9	4
III/T_3_N_0(1)_M_0_	7	3

Total	23	12

**Table 2:** List of chromosome 3 NotI-sites with methylation/deletion frequencies more than 17% in clear cell renal cell carcinoma.

Number	NotI-site*	Gene**	Locus	Methylation/deletion, frequency %
1	NL1-CJ4R (C)	*NKIRAS1/RPL15 *	3p24.2	57 (13/23)
2	NL6-FJ5R (C)	*LRRN1 *	3p26.2	43 (10/23)
3	NL3-CA11RS	*LRRC3B *	3p24	43 (10/23)
4	NLJ-003RD	*RBSP3 *(*CTDSPL*)	3p21.3	35 (8/23)
5	NL3003R (U)	*GORASP1/TTC21A *	3p22–p21.33	35 (8/23)
6	NRLA404R (U)	*VHL *	3p25.3	30 (7/23)
7	NR1-XM13C	*IQSEC1 *	3p25.2	26 (6/23)
8	NL1-BA6R	*FOXP1 *	3p14.1	26 (6/23)
9	NR1-AN24RS	*ABHD5/C3orf77 *	3p21	22 (5/23)
10	NL3A006R (D)	*NBEAL2 *	3p21.31	22 (5/23)
11	NL3A001R (D)	*GNAI2 *	3p21.31	22 (5/23)
12	NR1-NC7RS	*PPM1M *	3p21.2	22 (5/23)
13	NR1-NJ9R (C)	*PRICKLE2 *	3p14.1	22 (5/23)
14	HSJ4-AB7R (C)	*RPL32/IQSEC1 *	3p25.2	17 (4/23)
15	NL4-DP2RS	*FGD5 *	3p25.1	17 (4/23)
16	NL4-AP18R (C)	*PLCL2 *	3p24.3	17 (4/23)
17	NL4-BC8R (C)	*ALDH1L1 *	3q21.3	17 (4/23)
18	NL1A079R (D)	*EPHB1 *	3q21–q23	17 (4/23)
19	NR1-PD1R	*ZIC4 *	3q24	17 (4/23)

*Note*: *sequences are available at http://www.ncbi.nlm.nih.gov/nuccore/;**slash between gene names indicates that these genes have common NotI-site.

**Table 3:** Frequency of alterations and relative mRNA level of 8 genes in ccRCC and pRCC.

Genes	Frequency of mRNA level changes, %				Median of mRNA level changes, *n*-fold*	
	ccRCC		pRCC		ccRCC	pRCC
	↓	↑	↓	↑		
*LRRN1 *	**53** (9/17)	0 (0/17)	11 (1/9)	33 (3/9)	3↓(1–15↓)	3↑(2.1 × 10^3^↑–10↓)
*GORASP1 *	**42** (5/12)	0 (0/12)	36 (4/11)	0 (0/12)	1 (1–3↓)	1 (1–7↓)
*IQSEC1 *	0 (0/12)	8 (1/12)	**58** (7/12)	0 (0/12)	1 (3↑–1)	2↓ (1–53↓)
*FOXP1 *	20 (2/10)	0 (0/10)	17 (2/12)	25 (3/12)	1 (1–2↓)	1 (19↑–14↓)
*GNAI2 *	8 (1/12)	33 (4/12)	17 (2/12)	25 (3/12)	1 (4↑–2↓)	1 (3↑–4↓)
*FGD5 *	25 (3/12)	8 (1/12)	**83** (10/12)	0 (0/12)	1 (3↑–7↓)	3↓(1–59↓)
*PLCL2 *	**50** (6/12)	0 (0/12)	**83** (10/12)	8 (1/12)	1 (1–5↓)	3↓(2↑–8↓)
*ALDH1L1 *	**92** (11/12)	0 (0/12)	**92** (11/12)	0 (0/12)	4↓(1–1.1 × 10^2^↓)	6↓(1–1.6 × 10^2^↓)

*Note*: qPCR data. ↓/↑: mRNA level decrease/increase. *In parentheses a range of relative mRNA level is shown. The highest frequencies of mRNA level changes are shown in bold (*P* < 0.05 for all cases).
